# Retraction: The PU.1-Modulated MicroRNA-22 Is a Regulator of Monocyte/Macrophage Differentiation and Acute Myeloid Leukemia

**DOI:** 10.1371/journal.pgen.1012224

**Published:** 2026-07-09

**Authors:** The PLOS Genetics Editors

After this article [1] was published, concerns were raised regarding results presented in Figs 4, 6, and S3.

Specifically:

- The following panels appear similar:
  - Fig 4C THP1 MECOM Scr miR-22 panel in [1] and Fig 4G PIK3CD panel in [2, retracted in 3]
  - Fig 4C THP1 GAPDH Scr miR-22 panel in [1], Fig 4G GAPDH panel in [2, retracted in 3], and Fig 6c GAPDH in [4]
  - Fig 6A GAPDH lanes 1-4 in [1], Fig 2f GAPDH in [5], and Fig 6a GAPDH panel for CD34+HSPCs in [5]
- The following panels in [1] appear similar, despite representing different experimental conditions:
  - Fig 4C HL60 GAPDH Scr miR-22 and Fig S3 GAPDH Scr miR-22
  - Fig 4C HL60 MECOM Anti-Con Anti-miR-22 and Fig S3 MECOM Anti-con Anti-miR-22
  - Fig 6B GAPDH Lenti-ZIP-Con Lenti-ZIP-miR-22 and Fig 6D GAPDH

The corresponding author provided the available underlying data for Fig 4C and stated that these data were generated in the corresponding author’s lab by the first author in 2013, and that they did not share any raw data, images, or experimental materials with the authors of [2,4]. However, their explanation did not resolve the journal's concerns about the similarity of these data with results presented in [2,4] which were submitted before this article [1] was published, and the data provided did not resolve the concerns about the Fig 4C THP1 MECOM Scr miR-22 and THP1 GAPDH Scr miR-22 panels.

The corresponding author stated that the Fig 6A GAPDH lanes 1–4 in [1], Fig 2f GAPDH in [5], and Fig and Fig 6a GAPDH panel for CD34 + HSPCs in [5] are from the same blot, that these figures show the same samples and conditions, and they provided the blot data underlying Fig 6A.

Regarding the above-described similarities between Fig 4C and Fig S3 in [1], the corresponding author stated that the panels in Figs 4C and Fig S3 represent the same samples treated under the same conditions, and that the caption of Fig S3 is incorrect as these samples were not treated with PMA. Regarding the above-described concerns for the Fig 6B and Fig 6D panels, the corresponding author stated that Fig 6B is incorrect and provided a replacement panel.

In light of the above unresolved concerns that question the reliability and integrity of the reported results in [1], the *PLOS Genetics* Editors retract this article.

CS, MTC, XHZ, XLY, HMN, HSL, LS, FW, YNM, HLZ, JY, and JWZ did not agree with the retraction and stand by the article’s findings. RS either did not respond directly or could not be reached.

In addition to the above issues, after publication of this article [1]:

- The corresponding author stated that the sample size in the Fig 1A BMNC panel for the AML group (n = 41) is incorrect and should instead read n = 37.
- It was noted that Fig 4C THP1 MECOM Anti-Con Anti-miR-22 in [1] appears similar to Fig 3A N-cadherin in a later article by different authors [6, retracted in 7]. The corresponding author stated that they did not share any raw data, images, or experimental materials with the authors of [6].

## References

[1] Shen C, Chen M-T, Zhang X-H, Yin X-L, Ning H-M, Su R, et al. RETRACTED: The PU.1-Modulated MicroRNA-22 Is a Regulator of Monocyte/Macrophage Differentiation and Acute Myeloid Leukemia. PLoS Genet. 2016;12(9):e1006259. doi: 10.1371/journal.pgen.1006259 27617961 PMC5019412

[2] Yu L, Gong X, Sun L, Zhou Q, Lu B, Zhu L. The Circular RNA Cdr1as Act as an Oncogene in Hepatocellular Carcinoma through Targeting miR-7 Expression. PLoS One. 2016;11(7):e0158347. doi: 10.1371/journal.pone.0158347 27391479 PMC4938625

[3] The *PLOS ONE* Editors. Retraction: The Circular RNA Cdr1as Act as an Oncogene in Hepatocellular Carcinoma through Targeting miR-7 Expression. PLoS One. 2023;18(3):e0283112. doi: 10.1371/journal.pone.0283112 36989242 PMC10057760

[4] Sun L, Liang J, Wang Q, Li Z, Du Y, Xu X. MicroRNA-137 suppresses tongue squamous carcinoma cell proliferation, migration and invasion. Cell Prolif. 2016;49(5):628–35. doi: 10.1111/cpr.12287 27571935 PMC6495205

[5] Chen M-T, Dong L, Zhang X-H, Yin X-L, Ning H-M, Shen C, et al. ZFP36L1 promotes monocyte/macrophage differentiation by repressing CDK6. Sci Rep. 2015;5:16229. doi: 10.1038/srep16229 26542173 PMC4635361

[6] Liu X, Yidayitula Y, Zhao H, Luo Y, Ma X, Xu M. RETRACTED ARTICLE: LncRNA LINC00152 promoted glioblastoma progression through targeting the miR-107 expression. Environ Sci Pollut Res Int. 2018;25(18):17674–81. doi: 10.1007/s11356-018-1784-x 29671226

[7] Liu X, Yidayitula Y, Zhao H, Luo Y, Ma X, Xu M. Retraction Note: LncRNA LINC00152 promoted glioblastoma progression through targeting the miR-107 expression. Environ Sci Pollut Res Int. 2024;31(31):44449. doi: 10.1007/s11356-024-34118-8 38918299

